# Methanotrophs: Discoveries, Environmental Relevance, and a Perspective on Current and Future Applications

**DOI:** 10.3389/fmicb.2021.678057

**Published:** 2021-05-14

**Authors:** Simon Guerrero-Cruz, Annika Vaksmaa, Marcus A. Horn, Helge Niemann, Maite Pijuan, Adrian Ho

**Affiliations:** ^1^Catalan Institute for Water Research (ICRA), Girona, Spain; ^2^Universitat de Girona, Girona, Spain; ^3^Department of Marine Microbiology and Biogeochemistry, NIOZ Royal Netherlands Institute for Sea Research, ’t Horntje, Netherlands; ^4^Institute of Microbiology, Leibniz Universität Hannover, Hannover, Germany; ^5^Department of Earth Sciences, Faculty of Geosciences, Utrecht University, Utrecht, Netherlands; ^6^Centre for Arctic Gas Hydrate, Environment and Climate, Department of Geosciences, UiT the Arctic University of Norway, Tromsø, Norway; ^7^Division of Applied Life Sciences, Gyeongsang National University, Jinju, South Korea

**Keywords:** methanotrophy, application, methane, resource recovery, microbial ecology, climate change, anaerobic, circular economy

## Abstract

Methane is the final product of the anaerobic decomposition of organic matter. The conversion of organic matter to methane (methanogenesis) as a mechanism for energy conservation is exclusively attributed to the archaeal domain. Methane is oxidized by methanotrophic microorganisms using oxygen or alternative terminal electron acceptors. Aerobic methanotrophic bacteria belong to the phyla Proteobacteria and Verrucomicrobia, while anaerobic methane oxidation is also mediated by more recently discovered anaerobic methanotrophs with representatives in both the bacteria and the archaea domains. The anaerobic oxidation of methane is coupled to the reduction of nitrate, nitrite, iron, manganese, sulfate, and organic electron acceptors (e.g., humic substances) as terminal electron acceptors. This review highlights the relevance of methanotrophy in natural and anthropogenically influenced ecosystems, emphasizing the environmental conditions, distribution, function, co-existence, interactions, and the availability of electron acceptors that likely play a key role in regulating their function. A systematic overview of key aspects of ecology, physiology, metabolism, and genomics is crucial to understand the contribution of methanotrophs in the mitigation of methane efflux to the atmosphere. We give significance to the processes under microaerophilic and anaerobic conditions for both aerobic and anaerobic methane oxidizers. In the context of anthropogenically influenced ecosystems, we emphasize the current and potential future applications of methanotrophs from two different angles, namely methane mitigation in wastewater treatment through the application of anaerobic methanotrophs, and the biotechnological applications of aerobic methanotrophs in resource recovery from methane waste streams. Finally, we identify knowledge gaps that may lead to opportunities to harness further the biotechnological benefits of methanotrophs in methane mitigation and for the production of valuable bioproducts enabling a bio-based and circular economy.

## Introduction to Methane and Methane Microbiology

### Methane

Methane represents the most reduced form of carbon, is an important fuel for the global economy, and a greenhouse gas (GHG) in the atmosphere. It has a higher heat retentive capacity compared to CO_2_, estimated at 34 times higher in a 100-year timeframe and 86 times higher in a period of 20 years [Intergovernmental Panel on Climate Change (IPCC), 2018]. Furthermore methane concentration has been continuously increasing to approximately 1857 ppb in 2018, 2.6 times higher than in the preindustrial times (Saunois et al., 2020) making it a critical environmental concern for climate change (Myhre et al., 2013; Etminan et al., 2016; Dean et al., 2018).

Methane originates from abiogenic, thermogenic and biogenic microbial sources. In this work, we focus on biogenic methanogenesis by methanogenic archaea; which liberate methane as the end-product from biological decomposition of organic matter as a mean of energy conservation (McInerney and Bryant, 1981; Conrad, 2009; Drake et al., 2009). Methanogenic archaea are present in diverse environments such as wetlands, peatlands, rice agriculture soil, livestock (enteric fermentation in ruminants), landfills, oceans and termites; representing approximately 70% of all sources of methane emissions to the atmosphere (Conrad, 1996; Kirschke et al., 2013). Among these methane sources, agriculture (e.g., rice paddy fields), livestock farming, waste and wastewater treatment (WWT), and fossil fuels are subject to significant human-driven intensification, linked to an expanding global population that demands for supply and in return produce more waste and increase emissions [Intergovernmental Panel on Climate Change (IPCC), 2015; Wolf et al., 2017].

**FIGURE 1:**
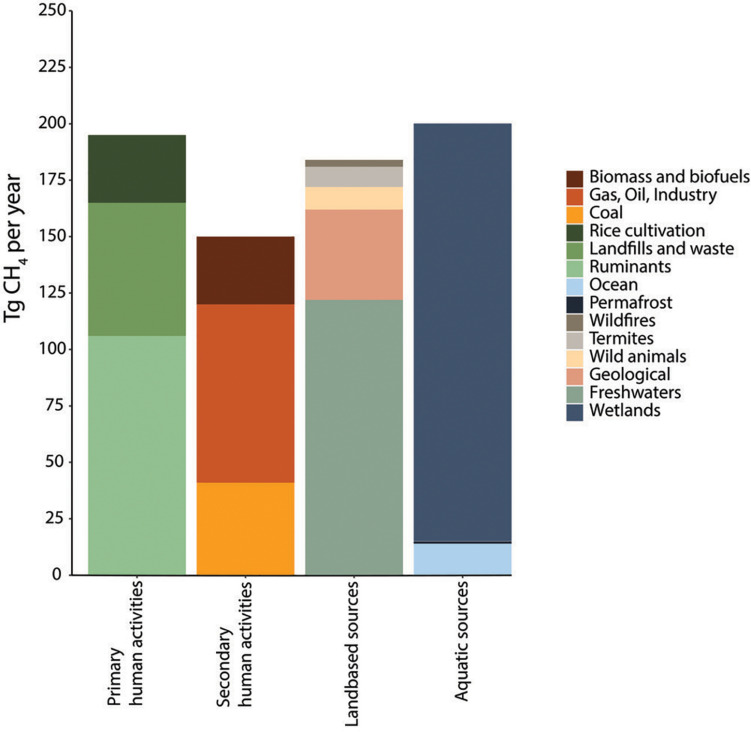
Methane emissions from 2008 to 2017, using a bottom-up approach (based on Saunois et al., 2020). Only average values are shown. The category “Human primary activities” is the focus of this review, methanotrophs receive special attention in wastewater treatment regarding their potential to mitigate GHG emissions and valorization of waste (Sections “Fate of Methane and Application Potential of Anaerobic Methanotrophs in Wastewater Treatment” and “Applications of Aerobic Methanotrophs in a Circular Economy”).

Most recent estimations from data over the past decade (2008–2017) indicate a total average net methane production of 737 Tg CH_4_ year^–1^ from all sources (ranging from 594 to 881 Tg CH_4_ year^–1^) (Figure 1), whereas total terrestrial and aquatic sinks are estimated at an average of 625 Tg CH_4_ year^–1^ (ranging from 500 to 798 Tg CH_4_ year^–1^) leaving a positive net average balance of 112 Tg CH_4_ year^–1^ escaping into the atmosphere (Saunois et al., 2020). However, the net emissions may be underestimated because of methane emissions from the novel and environmentally damaging practice of shale gas extraction (fracking) (Umeozor et al., 2018; Howarth, 2019). Similarly, estimations of methane emissions from wastewater treatment are scarce and accurate determinations are limited (Nguyen et al., 2019) (in detail in Section “Fate of Methane and Application Potential of Anaerobic Methanotrophs in Wastewater Treatment”). In addition, methane estimations are likely to vary significantly with increasing global temperatures, making the estimations, control and mitigation of methane of crucial importance to control global warming (Collins et al., 2018). From all anthropogenic sources, livestock, waste and WWT, and rice cultivation represent approximately 57% of the total anthropogenic methane emissions indicating the need for better understanding and development of methane mitigation strategies and the microbial processes that regulate the methane cycle (Conrad, 2009). Interestingly, methane has been postulated as the most cost-effective carbon feedstock for microbial chemical production, surpassing the cost competitiveness of chemical methanol or glucose from plant-derived sources. This opens opportunities for the application of methanotrophs to counteract methane emissions while producing valuable compounds derived from their metabolism (Section “Applications of Aerobic Methanotrophs in a Circular Economy”) (Comer et al., 2017). The following sections provide an overview of both methanogenesis and methanotrophy as microbial processes regulating methane fluxes worldwide.

### Methanogenesis

During the degradation of organic matter, commonly referred as anaerobic digestion (AD), a limited number of archaeal groups are known to produce methane in the final stage of AD, namely methanogenesis, using CO_2_, hydrogen, acetate, or methylated compounds as substrates coupled to the generation of energy for growth and carbon fixation (Liu, 2010; Sieber et al., 2010; Conrad, 2020). Despite substrate variation, methanogenesis is in all cases carried out through the action of the Methyl coenzyme M reductase (McrA) enzyme (Thauer, 1998).

Until very recently, only seven orders from the Euryarchaeota phylum were recognized as methanogenic archaea: Methanobacteriales, Methanocellales, Methanococcales, Methanomicrobiales, Methanomassiliicoccales, Methano- sarcinales, and Methanopyrales (Liu, 2010; Enzmann et al., 2018). Today, it is known that putative methanogenic archaea are spread beyond the Euryarchaeota. Through the application of in-depth genomic sequencing the classification and diversity of methanogenic archaea has rapidly evolved. Currently, methanogenic archaea have representatives in 4 recognized superphyla: Euryarchaeota, TACK (Thaumarchaeota, Aigarchaeota, Crenarchaeota, and Korarchaeota), DPANN (Diapherotrites, Parvarchaeota, Aenigmarchaeota, Nanoarchaeota, and Nanohaloarchaeota), and the Asgard superphylum (Evans et al., 2015; Lloyd, 2015; Vanwonterghem et al., 2016; Castelle and Banfield, 2018; Zhou et al., 2018; Berghuis et al., 2019; Dombrowski et al., 2019; Macleod et al., 2019).

### Methanotrophy

**FIGURE 2:**
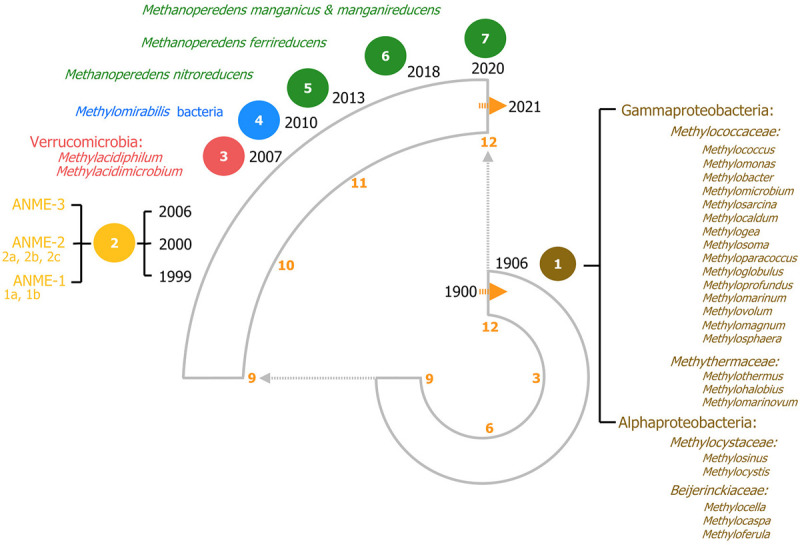
120 years of methanotrophy depicted in a clock-based timeline, each hour on the clock represents one decade. The most relevant findings are depicted highlighting all recognized methanotrophic groups categorized according to their phylogenetic classification. Seven major milestones in methanotrophy are included, representing the most noteworthy microbial discoveries. (1) In brown, the discovery of aerobic canonical methanotrophy in 1906. On the right, all the known genera according to phylogenetic classification are listed (based on Dedysh and Knief, 2018). (2) In yellow, the discovery of sulfate-dependent anaerobic oxidation of methane (S-dAOM). This process is performed by three defined groups of AN-aerobic ME-thanotrophic archaea (ANME) within the Euryarchaeota phylum. (3) In red, aerobic methanotrophy within the Verrucomicrobia phylum. (4) In blue, nitrate-dependent anaerobic oxidation of methane (N-dAOM) by *Methylomirabilis* bacteria. (5–7) In green, nitrate-, iron-, and manganese-dependent anaerobic oxidation (N-dAOM and Metal-dAOM) of methane by diverse cultured species of the *Methanoperedenaceae* family.

Methanotrophic microorganisms oxidize methane to harness energy under oxic and anoxic conditions using a range of diverse electron acceptors. Methanotrophy was initially reported in 1906 as an oxygen-dependent process and for almost a century, aerobic methanotrophy was considered as the only biological pathway to oxidize methane and that all methanotrophs belonged to the Proteobacteria phylum (Figure 2; Whittenbury et al., 1970; Hanson and Hanson, 1996). However, discoveries in the last two decades have broadened the view of methanotrophy with the identification of microorganisms outside the proteobacteria phylum and even in the archaea domain, capable of oxidizing methane anaerobically using alternative electron acceptors such as sulfate, nitrite, nitrate, iron and manganese (Figure 2). In the next sections aerobic and anaerobic methanotrophy and metabolic pathways are described in chronological order.

#### Aerobic Methanotrophs

Aerobic methane oxidation is catalyzed by particulate and soluble methane monooxygenases (pMMO and sMMO, respectively). These enzymes oxidize methane to methanol, and subsequently, methanol dehydrogenases (MDHs) further oxidize methanol to formaldehyde (Keltjens et al., 2014). After formaldehyde, two more oxidation steps are involved and the intermediates are used for carbon assimilation, for a detailed description on methanotrophy see Dedysh and Knief (2018). Aerobic methanotrophs belong to the Gammaproteobacteria (Type I, with families *Methylococcaceae* and *Methylothermaceae*), Alphaproteobacteria (Type II, with families *Methylocystaceae* and *Beijerinckiaceae*), and the Verrucomicrobia phyla (family *Methylacidiphilaceae*) (Figure 2, based on van Teeseling et al., 2014; Dedysh and Knief, 2018). Verrucomicrobial methanotrophs are likely to play a critical role in methane oxidation in extreme environments where they were initially discovered (i.e., geothermal and volcanic environments) (Dunfield et al., 2007; Pol et al., 2007; Islam et al., 2008), whereas proteobacterial methanotrophs are also found and active in environments with extreme conditions besides volcanic soils (i.e., acidic wetlands, thermal springs, thermal lakes, and peat soils) (Kolb and Horn, 2012; Islam et al., 2020, 2021; Kaupper et al., 2020; Hogendoorn et al., 2021). While most aerobic methanotrophs possess the pMMO, *Methylocella* and *Methyloferula* (Alphaproteobacteria, Beijerinckiaceae) harbor only the sMMO (Dedysh and Dunfield, 2016). Proteobacterial methanotrophs can be distinguished based on their taxonomy, carbon metabolism, morphology, and ecological life strategies among other features (Hanson and Hanson, 1996; Ho et al., 2013a; Knief, 2015; Dedysh and Knief, 2018; Hakobyan and Liesack, 2020). All pMMO-containing methanotrophs use copper as the catalytic metal in the first step of methane oxidation to methanol; in methanotrophs harboring both the pMMO and sMMO, copper suppresses the expression of the sMMO (Knapp et al., 2007; Trotsenko and Murrell, 2008) which require iron as they belong to a diverse enzymatic family of di-iron carboxylate enzymes (sDIMO) (Crombie and Murrell, 2014; Nichol et al., 2019). Strikingly, Verrucomicrobial methanotrophs and specific proteobacterial methanotrophs (e.g., *Methylocella*) rely on lanthanide metals for the second step of methanol oxidation as the catalytic driving force in MDHs from the XoxF family (Pol et al., 2014; Farhan Ul Haque et al., 2019; Smith and Wrighton, 2019). For a detailed review on the diversity of sDIMO methanotrophs, see Farhan Ul Haque et al. (2020).

#### Anaerobic Methanotrophs

The Anaerobic Oxidation of Methane (AOM) was first observed in marine sediments where methane consumption was linked to the presence of sulfate (Knittel and Boetius, 2009). However, the biology of this process was never fully characterized likely due to the lack of advanced cultivation and genomic technologies; furthermore, archaea were not yet discovered at the time thus a potential consortium of bacteria was hypothesized (Reeburgh, 1976; Reeburgh and Heggie, 1977; Zehnder and Brock, 1980). Two decades later, with the reclassification of living organisms (Woese and Fox, 1977; Fox et al., 1977), archaea were identified as the most likely cause for methane consumption based on carbon isotope studies (Hinrichs et al., 1999). Shortly after, the microbiology behind this process was described as sulfate-dependent anaerobic methane oxidation process (S-dAOM) by a microbial consortium consisting of sulfate-reducing bacteria (SRB) and methanotrophic archaea (Boetius et al., 2000). The identity of the Anaerobic Methanotrophic archaea (ANME) was confirmed soon after, currently classified as ANME 1, 2, 3 (Figure 2; Hinrichs et al., 1999; Boetius et al., 2000; Orphan et al., 2001; Niemann et al., 2006). The mechanism behind the oxidation of methane was identified as a reverse reaction of the canonical final step in the methanogenesis pathway (Krüger et al., 2003; Hallam et al., 2004). Interestingly, the reversibility of the last step of methanogenesis can also be encountered in canonical methanogens where iron minerals are known to stimulate methanotrophy in methanogens (Bar-Or et al., 2017). Furthermore, model methanogens such as *Methanosarcina barkeri* are known to oxidize methane through extracellular electron transfer (EET), referred as electrogenic anaerobic methane oxidation (Yu et al., 2021).

**FIGURE 3:**
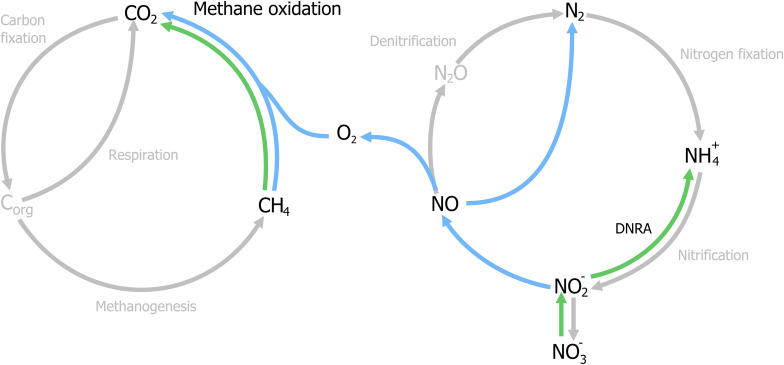
Schematic representation of the nitrate- and nitrite-dependent Anaerobic Oxidation of Methane (N-dAOM). *Methylomirabilis* bacteria (process in blue) reduce nitrite and through an intra-aerobic pathway, oxidize methane to CO_2_. “*Ca. M. nitroreducens*” (process in green) reduce nitrate, with nitrite as an intermediate, while oxidizing methane anaerobically using nitrate as terminal electron acceptor. Furthermore, *Methanoperedens* archaea are capable of producing ammonia through dissimilatory nitrate reduction (DNRA).

With the identification of archaea as the responsible microorganisms for the S-dAOM process in marine ecosystems, it became evident that methanotrophy is possible with alternative electron acceptors besides oxygen. In freshwater ecosystems, nitrogen oxyanions such as nitrite and nitrate are more abundant than sulfate, and thermodynamically suitable to support other microbial processes using methane as the energy source, a process commonly referred as nitrogen-dependent anaerobic methane oxidation (N-dAOM) (Reeburgh and Heggie, 1977; In ‘t Zandt et al., 2018a). After initial indications of methane oxidation occurring under anoxic conditions coupled to denitrification in WWT (Sollo et al., 1976), a microbial consortium of bacteria from the novel NC10 phylum (Holmes et al., 2001) and archaea from the former ANME-2d group performing this process (currently *Methanoperedenaceae*), was identified from anoxic sediments in agricultural soil (Raghoebarsing et al., 2006). The bacterium from the NC10 phylum, *“Candidatus Methylomirabilis oxyfera”* (Figure 2) was identified as the microorganism capable of oxidizing methane at the expense of nitrite as electron acceptor (nitrite-dAOM) (Ettwig et al., 2008). *“Ca. M. oxyfera”* harbored all the genes necessary to perform conventional aerobic methane oxidation via pMMO and MDH enzymes (Ettwig et al., 2010). The presence of these genes was inconsistent for a bacterium of anaerobic nature and highly susceptible to oxygen, it was then postulated that oxygen was formed from the dismutation of nitric oxide produced from nitrite reduction, this oxygen produced intracellularly, would then be used to oxidize methane via an intra-oxygenic pathway (Figure 3) (Wu et al., 2011; Ettwig et al., 2012). To date, the accurate characterization of the putative nitric oxide dismutase enzyme remains elusive and limited to the detection of these enzymes in environmental samples and candidate genes in enrichment cultures (Wu et al., 2015; Zhu et al., 2017, 2020; Versantvoort et al., 2018). Furthermore, the relevance of lanthanides as an essential metal for the oxidation of methanol, extends into the NC10 phylum where lanthanide-dependent MDH enzymes are seemingly more important and widespread than previously thought (Wu et al., 2015). To date, all bacteria capable of performing nitrite-dAOM with a potential intra-oxygenic pathway belong to the *Methylomirabilis* genus of the NC10 phylum (Versantvoort et al., 2018). *Methylomirabilis* bacteria are widespread in diverse environments, including freshwater and marine ecosystems (He et al., 2015; Padilla et al., 2016; Vaksmaa et al., 2016; Graf et al., 2018; Thamdrup et al., 2019), and in engineered ecosystems such as wastewater treatment plants (WWTPs, Section “Fate of Methane and Application Potential of Anaerobic Methanotrophs in Wastewater Treatment”) (Ettwig et al., 2009).

The archaeal partner was identified as a member of the new *Methanoperedenaceae* family (formerly ANME-2d) from the Euryarchaeota phylum and named “*Candidatus Methanoperedens nitroreducens,”* capable of coupling the reduction of nitrate to ammonia with nitrite as intermediate, to the oxidation of methane (nitrate-dAOM) via the reverse last step from methanogenesis (Haroon et al., 2013). The intermediate production of nitrite during the reduction of nitrate to ammonia, has demonstrated to be the common link that enables both *“Ca. M. nitroreducens”* and *Methylomirabilis* bacteria to coexist in laboratory enrichments and their simultaneous detection from environmental samples (Figure 3; Vaksmaa et al., 2017; Gambelli et al., 2018). Furthermore, *“Ca. M. nitroreducens”* has been demonstrated to be capable of performing methane oxidation using iron and manganese (ferrihydrite and birnessite) in the absence of nitrate (Ettwig et al., 2016) while other novel members of the *Methanoperedenaceae* family *“Ca. M. ferrireducens,” “Ca. M. manganireducens,”* and *“Ca. M. manganicus”* (Figure 2), do so exclusively with iron and manganese lacking the necessary genetic traits to use nitrate as terminal electron acceptor (Cai et al., 2018b; Leu et al., 2020a). In all members of the *Methanoperedenaceae* family, a complex and abundant network of cytochromes for direct electron transfer is likely to enable methane oxidation (Leu et al., 2020b). After an overview on methanogenesis and methanotrophy, the next section focuses on the environmental prevalence and relevance of the methanotrophs.

## Environmental Prevalence and Relevance of Methanotrophs Under Microaerophilic and Anaerobic Conditions

The ubiquity of (an)aerobic methanotrophs is attributable to their metabolic versatility, thriving in broad temperature (4–95°C) and pH (1–8) ranges (Trotsenko and Khmelenina, 2002; Pol et al., 2007). Particularly, aerobic methanotrophs have been the focus of previous reviews covering environmental detection, prevalence, and relevance in widespread habitats, as well as potential applications (Semrau et al., 2010, 2018; Ho et al., 2013a; Knief, 2015; Strong et al., 2015; Kwon et al., 2019; Kalyuzhnaya et al., 2020). However, more recent evidence indicates that aerobic methanotrophs can thrive under oxygen-limitation, and even under anoxic conditions challenging the conventional view of aerobic methanotrophy. Here, we focused on this aspect to provide a novel perspective of aerobic methanotrophy in environments which may not usually support their physiological requirements. Furthermore, we discuss recent aspects of microbial ecology and novel physiological aspects of anaerobic methanotrophs.

### Aerobic Methanotrophs Under Micro-Oxic Conditions

The oxidation of methane to methanol by the MMO-containing methanotrophs requires molecular oxygen serving as the electron acceptor (Ross and Rosenzweig, 2017). Therefore, aerobic methanotrophs are typically active at oxic-anoxic interfaces where methane and oxygen gradients overlap (e.g., soil-overlaying water interface in rice paddies and peatlands; Reim et al., 2012; Kaupper et al., 2021). However, the methane-oxygen counter gradient is dynamic, and oxygen could be rapidly depleted by oxygen-respiring organisms. To facilitate methane oxidation under micro-oxic conditions, some aerobic gammaproteobacterial methanotrophs (e.g., *Methylobacter*) may possess high-affinity cytochromes (e.g., cytochrome *bd* ubiquinol-oxidoreductase), enabling methane oxidation at low oxygen levels in the nM range (Skennerton et al., 2015; Smith et al., 2018; Smith and Wrighton, 2019). The pMMO-containing aerobic methanotrophs also possess hemerythrin, a non-heme oxygen-binding protein acting as an oxygen scavenging transporter to the pMMO which appears to be a widespread feature among pMMO-containing aerobic methanotrophs (Kao et al., 2008; Rahalkar and Bahulikar, 2018). Hemerythrins, initially discovered in *Methylococcus capsulatus* Bath (gammaproteobacterial methanotroph; Kao et al., 2004), have since been detected in other aerobic methanotrophs (e.g., *Methylomicrobium alcaliphilum*; Kalyuzhnaya et al., 2013). The correlation between the expression of pMMO and hemerythrin (Chen et al., 2012) and the increase in hemerythrin expression under oxygen-limited conditions has been reported (Kalyuzhnaya et al., 2013; Yu et al., 2020). Strikingly, some gammaproteobacterial methanotrophs (e.g., *Methyloterricola, Methylomagnum, Methylomonas, Methylobacter, and Methylomicrobium*) encode relatively higher hemerythrins and hemerythrin-containing domains compared to than alphaproteobacterial methanotrophs (Rahalkar and Bahulikar, 2018). Numerous environmental studies employing stable isotope probing (SIP) or mRNA-based analyses, showed that gammaproteobacterial methanotrophs are predominant and thrive at oxic-anoxic interfaces with rapid oxygen fluctuations (e.g., lake sediments, mangrove sediments, rice paddies, and peatlands; He et al., 2012; Reim et al., 2012; Shiau et al., 2018; Kaupper et al., 2020, 2021). For instance, vertical stratification along an oxygen gradient in a stratified lake has been observed, supported by metagenomic analysis revealing the genetic potential for aerobic methane oxidation under micro-oxic conditions, with the gammaproteobacterial methanotrophs (e.g., *Methylobacter*) present at the oxic-anoxic interface, and alphaproteobacterial methanotrophs present and stratified at the oxic water layer (Blees et al., 2014; Rissanen et al., 2018, 2020).

Altogether, it seems aerobic methanotrophs are equipped to function under micro-oxic conditions albeit with altered metabolic capabilities. Moreover, under micro-oxic conditions, the gammaproteobacterial methanotroph *Methylomonas denitrificans* can couple methane oxidation to nitrate reduction as an energy conservation strategy, and releasing nitrous oxide (Kits et al., 2015). *Methylomonas sp*. has been detected in anaerobic cultures of nitrate-dAOM microorganisms, where the expression of the *pmoA* gene increased upon exposure to 5% oxygen_v/v_, highlighting the survival and latency of aerobic methanotrophs under anoxic methane/nitrate-rich conditions (Guerrero-Cruz et al., 2018). Besides nitrate, methane oxidation coupled to iron (in the form of ferrihydrite) and manganese reduction through direct electron transfer *via* cytochromes, or *via* an artificial organic electron acceptor (e.g., anthraquinone-2,6-disulfonate) have been implicated for both gammaproteobacterial (*Methylococcus*, *Methylomonas*) and alphaproteobacterial (*Methylosinus*) methanotrophs under hypoxia (Oswald et al., 2016; Tanaka et al., 2018; Zheng et al., 2020). Proteobacterial methanotrophs (e.g., *Methylomicrobium alcaliphilum* and *Methylocystis*-affiliated) excrete methane-derived organic compounds (e.g., acetate, lactate, succinate) during oxygen-limited growth (Costa et al., 2000; Kalyuzhnaya et al., 2013). The released organic compounds likely fueled and fostered a complex network of interacting microorganisms comprising both the methanotrophs and non-methanotrophs (He et al., 2020). A complex regulatory mechanism involving an interplay of carbon metabolism (methane and methanol oxidation), denitrification, quorum sensing, and production of secondary metabolite (tundrenone), mediated by the signaling molecules nitric oxide and oxygen has been implicated in the response of *Methylobacter* to hypoxia (Yu et al., 2020). The metabolic versatility exhibited by these aerobic methanotrophs may become advantageous traits in dynamic environments, with rapidly fluctuating oxygen availability. In addition, alphaproteobacterial methanotrophs (*Methylosinus* species) have been co-cultivated alongside strictly anaerobic methanogens under oxygen limitation (In ‘t Zandt et al., 2018b). Therefore, it is noteworthy that aerobic methanotrophs likely showed species- or even strain-specific metabolic versatility, if parallels are drawn from their N-metabolic capabilities (Hoefman et al., 2014), to cope under oxygen limitation.

Interestingly, *Methylobacter* has been detected to form an active methane-oxidizing community in prolonged dark-anoxic incubations of a sub-Arctic lake sediment, ruling out the possibility that photosynthetic organisms helped sustain aerobic methane oxidation (Martinez-Cruz et al., 2017). Nevertheless, aerobic methanotrophs can thrive in anoxic environments by closely associating with photosynthetic (micro) algae serving as oxygen source (van der Ha et al., 2011; Milucka et al., 2015). Rissanen et al. (2018), demonstrated that light stimulated methane oxidation by gammaproteobacterial methanotrophs, including *Methylobacter*, in the anoxic water layers of an oxygen-stratified lake. More recently, light-induced stimulation of aerobic methane oxidation was detected in a field-based study of a boreal lake (van Grinsven et al., 2021). Similarly, aerobic methanotrophs may also rely on Sphagnum for molecular oxygen, enabling their proliferation in anoxic niches in peatland ecosystems (Raghoebarsing et al., 2005; Ho and Bodelier, 2015); or independently relying on incomplete denitrification (van Grinsven et al., 2020). The interaction of aerobic methanotrophs with their biotic environment, appears to be relevant in modulating community functioning, and may help confer resilience during disturbances prompting the use of alternative strategies and the formation of synergistic/antagonistic interactions (Ho et al., 2014, 2016, 2020; Veraart et al., 2018; Chang et al., 2020; He et al., 2020; Kaupper et al., 2021). Here, such interactions expand the habitat range of the aerobic methanotrophs to encompass unexpected environments which usually do not meet the physiological requirements for aerobic methane oxidation.

### Anaerobic Methanotrophs, Physiology Dictates Prevalence

#### Environmental Studies

Anaerobic methanotrophic archaea (ANME) differ vastly in regard to taxonomy (75–92% 16S rRNA similarity) (Knittel et al., 2005; Knittel and Boetius, 2009), cell shape and aggregation mode (Orphan et al., 2002), and even lipid composition (Chevalier et al., 2014). ANME archaea performing S-dAOM are responsible for 80% of methane oxidation in marine ecosystems where sulfate concentrations are abundant (Hinrichs and Boetius, 2002; Boetius and Wenzhöfer, 2013). Recently, the versatility of ANME archaea has expanded by their use of humic substances, natural components of organic matter with redox-active functional groups such as quinone moieties, that could enable Humic-dAOM. Several studies have used the humic acid analog anthraquinone-2,6-disulfonic acid (AQDS) to demonstrate Humic-dAOM (Coates et al., 1998; Cervantes et al., 2000). Humic-dAOM has been observed in ANME2a and ANME2c in the presence of humic acids and AQDS as electron acceptors in the presence of chelated ferric iron (Scheller et al., 2016). Furthermore, humic substances were shown to stimulate methane oxidation linked to N_2_O reducing microbes in coastal sediments in microbial communities composed by Acinetobacter and archaea from the Rice Cluster I and an uncultured member of the *Methanomicrobiaceae* (Valenzuela et al., 2020).

Regarding N-dAOM microorganisms, environmental surveys yielded simultaneous detection of both *Methylomirabilis* bacteria and *“Ca. M. nitroreducens”* in a widely diverse range of environments such as, sandy riverbeds (Shen et al., 2019), lake sediments (Lomakina et al., 2020), stratified lakes (Deutzmann et al., 2011, 2014; Graf et al., 2018), various wetlands including peatlands, paddy fields swamps, canals and groundwater (Raghoebarsing et al., 2006; Zhu et al., 2012, 2015; Vaksmaa et al., 2016), estuary and coastal sediments (Chen et al., 2014) among others. For a broad summary on environmental detection see Ding and Zeng (2021). Intriguingly, the first detection of *Methanoperedens* archaea came from the Gulf of Mexico (Mills et al., 2003, 2005); however, N-dAOM in the marine environment was long underestimated due to their low abundance. Recently *“Ca. M. nitroreducens”* and *“Ca. M. oxyfera,”* were identified in cold seeps and in gas hydrate-bearing sediments of the deep sea which suggests N-dAOM is an overlooked sink for methane in marine environments (Jing et al., 2020). However the availability of nitrate and nitrite in the marine environment is much lower than sulfate, and is dependent on the fluxes of nitrogen deposition or degradation of organic matter present in the sediments.

Iron (Fe) as the most abundant element on earth by mass, is widely spread in marine (Beal et al., 2009; Wankel et al., 2012; Egger et al., 2015) and freshwater environments (Sivan et al., 2011; Nordi et al., 2013) indicating the potential environmental relevance of *Methanoperedens* archaea capable of Fe-dAOM (Aromokeye et al., 2020). In this sense, microbial ^13^C carbon assimilation into *Methanoperedens*-like archaea under iron-rich, sulfate-depleted sediment incubations, has been reported (Weber et al., 2017). In addition, sulfate-rich environments could support S-dAOM mediated by *Methanoperedens* archaea, with the possible involvement of iron^+3^ or manganese^+4^ according to geochemical evidence and the detection of these archaea in sulfate-rich freshwater lake Cadagno (Schubert et al., 2011). A follow-up study proposed S-dAOM to be carried out by *M. nitroreducens* in a consortium with Desulfobulbaceae (Su et al., 2020). The debate whether metal oxides alone can be used as electron acceptors or serve a stimulatory role to support S-dAOM, persists since no genome of the *Methanoperedens* archaea known to date, contains dissimilatory sulfate reductases (Leu et al., 2020b).

#### Laboratory Enrichments

Despite the widespread prevalence of AOM microbes in various environments, since their discovery, highly enriched laboratory cultures are very scarce and no pure culture has been reported, limiting physiological experiments. Through optimizations in bioreactor enrichments and cultivation media design, a limited number of cultures with enrichments from 20–83% of *Methanoperedens* archaea and/or *Methylomirabilis* bacteria, are available (Haroon et al., 2013; Guerrero-Cruz et al., 2018; Lu et al., 2018).

Mechanistic and enzymatic machinery to utilize diverse electron acceptors has been reported in several highly enriched laboratory cultures. The duality of *“Ca. M. nitroreducens”* to perform Nitrate-, Fe-, and Mn-dAOM in short term batch cultivations has been shown (Ettwig et al., 2016). However, metal mineral forms cannot be transported over cellular membranes, indicating the need for an extracellular electron transfer (EET) mechanism when no syntrophic partner is involved. *“Ca. M. nitroreducens”* genome encodes for a high number of multi-heme c-type cytochromes (Haroon et al., 2013; Arshad et al., 2015), which could facilitate the electron transfer, but to date, no direct conclusive intermediate interspecies electron transfer has been shown for *“Ca. M. nitroreducens.”* Other *Methanoperedens* archaea utilize iron and manganese in the absence of nitrate reducing genomic traits, where the involvement of cytochromes to mediate electron transfer was proposed based on genomic and transcriptomic experiments (Cai et al., 2018b; Leu et al., 2020a). In transcriptomes of *“Ca. M. manganicus”* and *“Ca. M. manganireducens,”* 23 of the 33, and 9 of the 19 multiheme c-type cytochromes were highly expressed, respectively. These studies propose Menaquinone as electron carrier based on the identification of menaquinone biosynthesis pathway in all the *Methanoperedens* strains. Comparative genomics of 16 genomes of *Methanoperedens* strains revealed that about 88% of genomes contained more than one of the menaquinone cytochrome oxidoreductase complexes and about 63% have at least four (Leu et al., 2020b).

An alternative mechanism for electron transfer is based on conductive nanowire structures (Leu et al., 2020a), similar to *“Ca. M. nitroreducens”* Mnv1 strain (Guerrero-Cruz et al., 2018), where upregulation of archaellum related genes was shown under oxidative stress. *“Ca. M. manganicus”* and *“Ca. M. manganireducens”* genomes encode for genes of the major subunit flagellin (flaB), part of the archaellum. Two of the four *flaB* genes were highly expressed during Mn-dAOM experiments in *“Ca. M. manganicus,”* suggesting the involvement of these conductive appendices in electron transfer. In addition to archaellum-mediated AOM, Humic-dAOM has been reported in an enrichment culture of both N-dAOM microorganisms where AQDS and anthraquinone-2-sulfonic acid (AQS) acted as electron acceptors for methane oxidation (Bai et al., 2019). These examples demonstrate with specific evidence the broad versatility of *Methanoperedens* archaea; however, other studies report AOM with alternative electron acceptors with inconclusive results. Hexavalent chromium (Lu et al., 2016), selenate (Luo et al., 2018; Shi et al., 2020), and bromate (Luo et al., 2017), have been suggested to mediate AOM in cultures containing N-dAOM microorganisms; however, the enrichment and reported activity of side populations of *Geobacter* or canonical aerobic methanotrophs, make the involvement of N-dAOM microorganisms, debatable.

Laboratory bioreactor systems have been fundamental in the discovery and isolation of important novel N- and metal-dAOM microorganisms, yet these systems alter the natural conditions thus opening the possibilities for mutations and induced evolution. Are these changes, natural overlooked features or are these features introduced *in vitro* thus altering natural occurring features? Mn-dAOM representatives, “*Ca. M. manganicus”* and *“Ca. M. manganireducens”* strains, were enriched (up to 50% enrichment) from parent pre-enriched inoculum of *“Ca. M. ferrireducens”* (25% enrichment) and a minimal proportion of “*Ca. M. manganicus”* (Leu et al., 2020a). Similarly, the nitrate-dAOM archaeon *“Ca. M. nitroreducens,”* has several strains described over the last 8 years since its original isolation and is the only member of the Methanoperedenaceae family encoding for *narGHI* genes necessary for nitrate reduction while having the capability to perform iron- and manganese-dAOM under nitrate-depletion (Haroon et al., 2013; Ettwig et al., 2016).

Similarly, *“Ca. M. oxyfera”* enrichments have been subject to adaptation triggered by laboratory conditions since its original isolation, as a result making correlations to natural environments challenging. Early reports established that *“Ca. M. oxyfera”* harbored the canonical aerobic methane oxidation pathways (Ettwig et al., 2010) including methanol dehydrogenase (MDH). MDH in *“Ca. M*. *oxyfera”* was reported as a heterodimer formed by Calcium-dependent *MxaI*-encoded small subunit and a *XoxF1*-encoded large subunit, furthermore, did not possess the complete PQQ synthesis genes, necessary for the coordination of methanol in the active site of MDH (Wu et al., 2015). XoxF-type enzymes are known to incorporate lanthanide metals in their active sites in order to catalyze the oxidation of methanol (Keltjens et al., 2014). Recent studies have shown that other species of *Methylomirabilis* bacteria have evolved under laboratory conditions from an *Methylomirabilis*-rich inoculum (Ettwig et al., 2009), cultivated with elevated amounts of Cerium. These conditions resulted in the enrichment of *“Ca. Methylomirabilis lanthanidiphila*,” the second highly enriched cultivated member of NC10 phylum, encoding only one XoxF-type MDH highlighting the potential involvement of lanthanides such as cerium in methanol oxidation (Versantvoort et al., 2018). In deep freshwater lakes, other species of *Methylomirabilis* bacteria have been identified harboring lanthanide-dependent MDH although yet uncultured (Graf et al., 2018). These recent findings highlight the MDH gene redundancy in the original *“Ca. M*. *oxyfera,”* if the role of Calcium-dependent MDH is a metabolic trait in the natural environment or triggered by enrichment conditions where lanthanides were not originally supplied, remains an open question.

Overall, quantification of the AOM rates linked to various electron acceptors is often based on laboratory batch incubations where a single process is investigated. At times, soils are preincubated to allow depletion of naturally occurring electron acceptors; this however, may affect the community and functioning of the original inoculum.

## Fate of Methane and Application Potential of Anaerobic Methanotrophs in Wastewater Treatment

The fate of methane relies on physical conditions such as gradient pressure, depth, and the presence and activity of the diverse array of methanotrophic microorganisms functioning as a biofilter. If the right conditions are met with availability of suitable electron acceptors, methanotrophs will consume methane before it escapes into the atmosphere. A perspective on the fate of methane focusing on the anaerobic methanotrophs and their role in human-influenced ecosystems is given, using wastewater treatment systems as a model.

### Carbon Removal in Wastewater Treatment

In wastewater (WW), pollutants are transported from households and/or industrial discharge into wastewater treatment plants (WWTPs). Since modern WWT developed in the late 1800s and formally implemented in the early 1900s, organic carbon removal is performed through microbial aerobic oxidation commonly known as activated sludge (AS) (Alleman and Prakasam, 1983). The AS process takes place in oxidation tanks requiring massive amounts of energy in the form of aeration through mechanical or pneumatic infrastructure. Organic matter (including sludge from AS), is also degraded through anaerobic digestion (AD), which is a sequential set of microbial conversions under reducing conditions in the absence of oxygen namely: hydrolysis, acidification, fermentation, and ultimately methanogenesis (Section “Methanogenesis”). The development of AD was halted for decades due to the lack of fundamental understanding of the microbiology behind methanogenesis at the time (Buswell and Hatfield, 1936; McCarty, 1982). In the 1980’s, researchers achieved the decoupling of the solid retention time (SRT) from the hydraulic retention time (HRT) with the implementation of granular growth. Through the implementation of fluidized bed reactors with granular biomass configuration, the Up-flow anaerobic sludge blanket reactor (UASB) became one of the golden standards in AD technology for full-scale applications (Lettinga et al., 1980; Lettinga and Hulshoff Pol, 1991; Lettinga, 1995). Today, the methane produced from AD in full-scale sewage treatment, is collected as a methane-rich gas mixture termed biogas with remaining environmental concerns (Section “Wastewater Treatment as a Source of Methane Emissions”). Biogas represents a sustainable use of waste for the production of electricity from methane and is one of the first circular economy examples of energy recovery from waste (Bachmann, 2015).

The complexity of the microbial communities in AD, the large volumes of wastewater, and the variations in organic matter load; pose particular disadvantages prompting to bioaugmentation protocols for biogas. Bioaugmentation is needed to remediate the variation in methane content, and the presence of toxic bioproducts such as hydrogen sulfide (Nzila, 2017; Okoro and Sun, 2019). Carbon removal either through AS or AD is a complex combination of microbial processes under highly concentrated nutrient load and accelerated enrichment conditions, however, the complexity of microbial populations remains vastly unexplored (Ho et al., 2013c; Wu et al., 2019). Only recently, global attempts to characterize WWT microbiology through sequencing technology, were initiated (Karst et al., 2018) aiming at the construction of novel taxonomy databases applied to WWT called MiDAS4 (Nierychlo et al., 2020) for the further understanding of microbial ecology in WWT including anaerobic digestion (AD). With these advancements, we anticipate that microbial engineering of AD will be unraveled and can provide custom-made solutions for waste degradation. This, combined with biotechnological production of tailored products from the wide array of resources contained in WW including volatile fatty acids (VFAs) and methane as a building blocks however, this possibility at the moment remains a black box in WWT engineering. Additionally, biogas represents a low-value and renewable feedstock for a sustainable and circular economy through the application of methanotrophs capable of producing valuable compounds and biopolymers from methane (Section “Applications of Aerobic Methanotrophs in a Circular Economy”) (Wang et al., 2020).

### Nitrogen Removal in Wastewater Treatment

**FIGURE 4:**
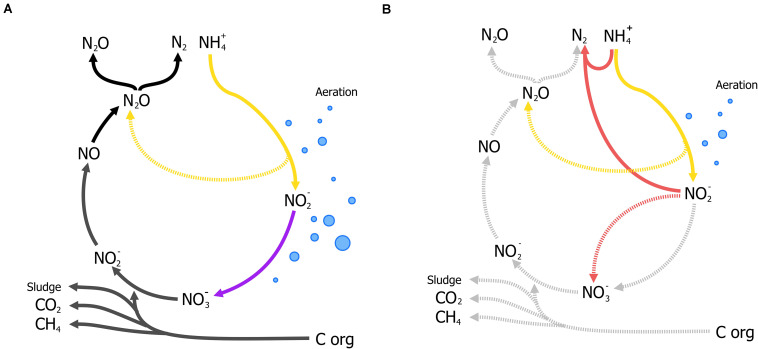
Traditional Nitrification/denitrification process (N/dN) **(A)** and anammox-mediated removal of ammonia coupled to partial nitritation **(B)**. **(A)** Shows the input and byproducts of the N/dN process: aeration (blue bubbles) for the complete oxidation of ammonia by aerobic ammonia oxidizers (AOB, in yellow) and nitrite oxidizing bacteria (NOB, in purple including complete ammonia oxidizers), organic carbon as electron donor for denitrification, and the production of sludge, and potential release of GHG gases (N_2_O) represented in dark gray. Potential emissions of N_2_O during nitrification (yellow, broken line) could take place under oxygen limitation (Caranto et al., 2016; Thakur and Medhi, 2019). **(B)** The partial-nitritation/anammox process (PN/A), where nitrite from partial-nitrification by AOB (in yellow) requiring less aeration (blue bubbles), is used to oxidize ammonia directly by anammox bacteria (in red), without performing the complete nitrification-denitrification (broken lines, light gray). Aeration control is crucial to balance nitrite production and the prevention of N_2_O from oxygen-limited nitrification.

Nitrogen as a form of pollution, is mainly represented by ammonia and has traditionally been removed through the canonical nitrification-denitrification process (N/dN). In N/dN, ammonia is oxidized to nitrate and subsequently reduced dinitrogen gas (N_2_) covering a large portion of the entire nitrogen cycle in one treatment process (Figure 4A). As in the activated sludge process, nitrification requires large amounts of energy in mixing and sufficient aeration to oxidize all nitrogen-compounds to nitrate. Furthermore, denitrification may require organic carbon (typically methanol) under poor carbon/nitrogen ratios as electron donors for the reduction steps from nitrate to N_2_ (Figure 4A). Moreover, under specific conditions nitrous oxide (N_2_O), a potent GHG, is produced and released to the atmosphere and current estimates report that 5% of the total N_2_O annual emissions come from WWT [Intergovernmental Panel on Climate Change (IPCC), 2014] (a full review on N/dN is given by Thakur and Medhi, 2019). The additional requirements for organic carbon, the large-scale tanks for N/dN processes, and the potential for GHG emissions, render this process unsustainable.

Analogously to the development of the N-dAOM, ammonia oxidation can also occur using nitrite as electron acceptor, a process known as nitrite-dependent anaerobic ammonia oxidation (anammox) performed by anammox bacteria from the phylum Planctomycetes, discovered and isolated from sewage sludge (Mulder et al., 1995; Van De Graaf et al., 1996; Strous et al., 1999a). Soon after their discovery, a series of fundamental physiology research studies enabled the anammox process to find technological applications to remove ammonia from wastewater. In full-scale applications, half of the ammonia is oxidized to nitrite by aerobic ammonia oxidizers (partial nitritation), which is then used by anammox bacteria to oxidize the remaining ammonia to N_2_, a combination known as partial nitritation/anammox (PN/A) (Figure 4B). The PN/A process offers significant advantages over N/dN and biologically, the anammox process represents a shortcut to the nitrogen cycle that has revolutionized ammonia removal worldwide (van der Star et al., 2007; Agrawal et al., 2018). For a full description of the historical developments of anammox, see Kuenen (2020).

### Wastewater Treatment as a Source of Methane Emissions

**FIGURE 5:**
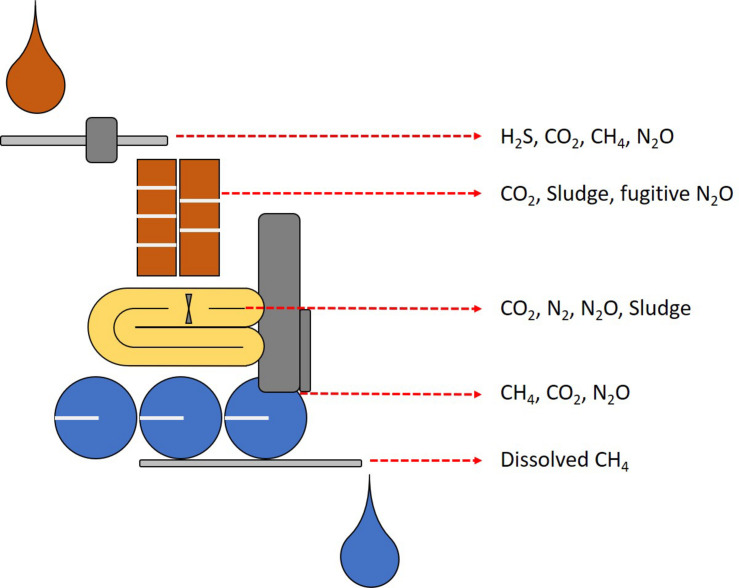
Schematic representation of the detrimental emissions from WWT. In brown, sewage transport and activated sludge systems produce nitrogen, sulfur and carbon gas emissions and large quantities of sludge. In yellow, canonical N/dN nitrogen removal systems can release nitrous oxide as the main concern, PN/A systems representing improvements in nitrogen removal (Figure 4). In grey, anaerobic digestion systems where primarily methane emissions take place from dissolved methane in effluents, and in blue, post treatment where dissolved emissions escape to the atmosphere and residual sludge is disposed with additional GHG emissions.

The need for innovative methane removal technologies originates because despite the significant advancements in AD and the possibility of recovering biogas for energy production from waste, some challenges remain. Waste and wastewater treatment technologies remain with open loops that call for novel microbiology-based technologies for GHG mitigation (Figure 5). Biological WWTPs, have received recent attention as a source of GHG (Weissenbacher et al., 2010; Daelman et al., 2012, 2013). In anaerobic sludge liquor, methane can be present at levels up to 26 grams per cubic meter (Chen et al., 2015) whereas other calculations from anaerobic systems predict up to 45% methane dissolved in effluents at maximum methane production capacity (Liu Z.H. et al., 2014). Effluents from WWTP, in particular from AD, carry a fraction of the methane dissolved which subsequently escapes to the atmosphere without control measures (Guisasola et al., 2008; Kampman et al., 2014; Schaum et al., 2016; Nguyen et al., 2019; Ribera-Guardia et al., 2019). Furthermore, in low-temperature waters, up to 60 per cent of methane can remain dissolved in the water and subsequently released into the atmosphere (Noyola et al., 2006; Souza et al., 2011).

With the evidence of methane emissions from WWT, current trends include attempts to estimate the contribution through the implementation of methodologies aiming to accurately determine the footprint of WWT (Guisasola et al., 2009; Rodriguez-Garcia et al., 2012). Current estimations indicate that WWT represents 2.8% of the total global GHG emissions, however the uncertainties in estimations range between 3% to 9% of total anthropogenic emissions (Stern and Kaufmann, 2014; Saunois et al., 2020). In the next section, an overview of the potential application of anaerobic methane and ammonia oxidation is given, emphasizing the research gaps needed to explore the possibilities of implementing a combined process where nitrogen (ammonia) removal and fugitive carbon emissions (methane) can be addressed simultaneously.

### Potential Applications of Anaerobic Methanotrophs in Wastewater Treatment

Wastewater treatment represents the ideal niche with the highest loads of nitrogen and carbon input suitable for N-dAOM methanotrophs and anammox to thrive. Anammox bacteria incorporated in PN/A systems are a clear example of a technology that developed from a novel microbial process characterized in laboratory-scale enrichments, and through physiology studies, reached full-scale realization (Van De Graaf et al., 1997; Strous et al., 1999b; Kuenen, 2020). Some of the microbial aspects relevant for the application of an anaerobic process in wastewater systems are growth, inhibition, and physiology; this knowledge is achieved through the combination of fundamental research on microbial physiology, engineering, genomics and even modeling (Strous et al., 1997; Carvajal-Arroyo et al., 2013; Lotti et al., 2014). Recently, the possibility to remove ammonia from WW coupled to electricity production was demonstrated for anammox bacteria (Shaw et al., 2020), capable of direct extracellular electron transfer (EET) similar to methanogens (Section “Anaerobic Methanotrophs”). EET anammox technology could potentially replace oxygen-limited systems such as PN/A circumventing the requirement for aeration, however this process is at early developmental stage in laboratory systems. EET in *Methanoperedens* archaea has been proposed as a decoupling mechanism from *Methylomirabilis* bacteria (Ding et al., 2017), however the reported enrichment is accompanied by the enrichment of canonical methanotrophs and geobacter under short term incubations. Furthermore, the implementation of EET-dAOM would require high quality methane supply based on the kinetic limitations of *Methanoperedens* archaea, e.g., in hollow membranes (Zhang et al., 2020) (Section “Methane Supply and Reactor Configuration”). For this purpose EET-dAOM is not further discussed.

**FIGURE 6:**
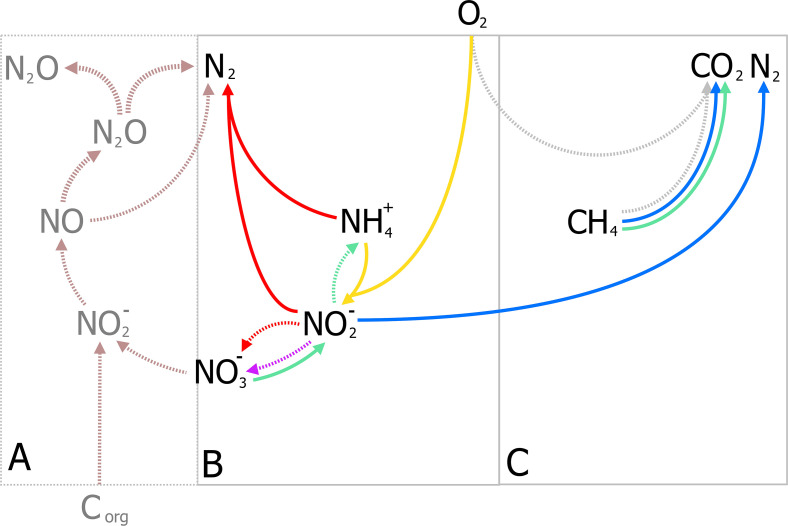
A schematic representation of the combination of partial-nitritation/anammox (PN/A) and N-dAOM processes in WWT. **(A)** Canonical denitrification where space, energy, and organic carbon, are needed for complete nitrogen removal in WWT. Denitrification produces sludge and GHG emissions as by-products under unfavorable conditions. **(B)** Partial-nitritation/anammox process, as a short cut to complete denitrification. AOB (in yellow) oxidize ammonia to nitrite, anammox bacteria (in red) oxidize ammonia anaerobically using nitrite as electron acceptor. **(C)** Incorporation of N-dAOM processes to PN/A, where both achieve the simultaneous removal of ammonia and methane (represented in green and blue for nitrate- and nitrite-dAOM, respectively). Broken arrows indicate competing processes that are deemed undesirable or processes that are circumvented by the implementation of the PN/A and N-dAOM processes. Adapted from Guerrero-Cruz (2018).

**FIGURE 7:**
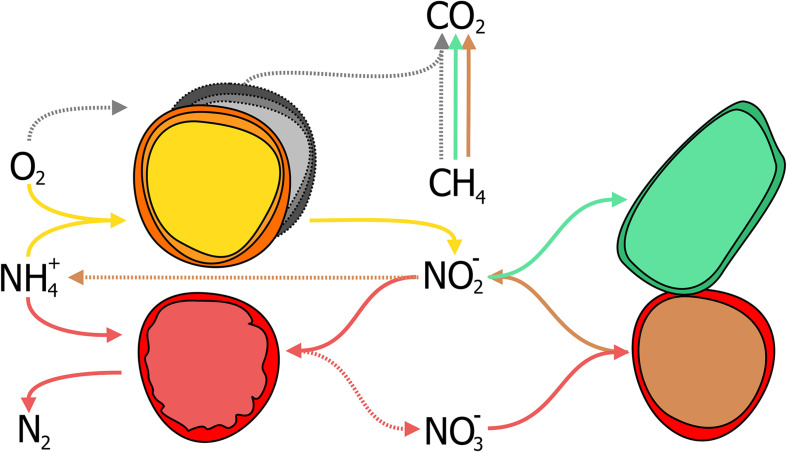
Interactions between anaerobic methane oxidizers performing N-dAOM with anaerobic ammonia oxidation (anammox) incorporated into partial-nitritation systems (PN/A). In existing PN/A systems for ammonia removal, aerobic ammonia oxidizers (AOB, in yellow) oxidize ammonia to nitrite, anammox bacteria (in red) use nitrite to oxidize ammonia to dinitrogen gas, while producing residual nitrate from carbon fixation. *“Ca. Methanoperedens nitroreducens”* (in brown with red outline) converts nitrate to nitrite as common intermediate while oxidizing methane to carbon dioxide and can produce ammonia. Nitrite either produced by AOB or *M. nitroreducens*, is used by *Methylomirabilis* bacteria (in green) for the oxidation of methane to carbon dioxide. Aerobic methane oxidizers (in grey) competefor oxygen with AOB, and can oxidize methane, in competition with anaerobic methane oxidizers.

Nitrogen-dependent anaerobic methane oxidation in WWT was considered a suitable application for simultaneous methane and nitrogen removal almost four decades ago (Davies, 1973; Sollo et al., 1976). Today, after the discovery of the microorganisms responsible for this process in diverse environments including WWT, and their enrichment in laboratory-scale reactors (Ettwig et al., 2009; Hu et al., 2009; Luesken et al., 2011b; Haroon et al., 2013), N-dAOM as a potential application has re-emerged (Wang et al., 2017). The N-dAOM process has significant potential benefits if the microorganisms responsible for this process can be integrated into oxygen-limited systems for ammonia removal (Figure 6). This concept is proposed based on laboratory scale bioreactor enrichments where both N-dAOM and anammox microorganisms co-exists with nitrite as a common link (Figure 7). Bioreactor systems supplied with nitrate, methane and ammonia; have resulted in a coculture of N-dAOM archaea, N-dAOM bacteria and anammox in diverse proportions (Shi et al., 2013; Stultiens et al., 2019). However, when supplied with nitrite, methane and ammonia only a combination of both bacterial partners (N-dAOM bacteria and anammox) was achieved (Luesken et al., 2011a). Based on these reports, models have been developed indicating that both anaerobic processes can coincide and could reach a practical application and performance in WWT (Winkler et al., 2015).

In short, the simultaneous removal of methane and ammonia in oxygen-limited systems relies in the concerted interaction of 4 microbial processes (Figure 6) driven by key microbial groups (Figure 7):

(i) Aerobic ammonia-oxidizing bacteria (AOB) oxidize half of the ammonia available to nitrite (partial nitritation, PN), requiring half the aeration energy compared to N/dN (Figure 6B).
(ii) Anammox bacteria, oxidize the remaining ammonia using the nitrite produced by AOB. Anammox however, produces nitrate as a side reaction during carbon fixation (Figures 4B, 6B).
(iii) Methane is oxidized by nitrite-dAOM bacteria using nitrite most likely under competition with anammox bacteria. Both producing nitrogen gas as final nitrogen-product (Figure 6C).
(iv) Nitrate-dAOM archaea, will compete with *Methylomirabilis* bacteria to oxidize methane using nitrate (Figure 6C). Nitrate can originate from anammox side reactions or undesirable complete nitrification of ammonia to nitrate or from other nitrate-rich effluents (Figure 6A).

Dissolved methane from diverse effluents, more importantly anaerobic digestion, and biogas mixtures that otherwise would not be used for electricity production; represent an ideal energy source for the combined process (Bandara et al., 2011; Wang et al., 2017), reaching up to 45% in some cases (Liu Z.H. et al., 2014). To successfully combine both processes, physiology and microbial ecology aspects are of vital importance. These aspects include competition for nitrite as an intermediate substrate, doubling times in a mixed culture, affinity and competition for methane by aerobic and anaerobic methane oxidizers, competition for oxygen between aerobic ammonia and methane oxidizing microorganisms (Figure 7) under oxygen-limited conditions, and more importantly the inhibition of anaerobic microorganisms upon the exposure to oxygen. Most of these microbial metabolic aspects are known for anammox bacteria (Strous et al., 1999b) but remain to be determined for N-dAOM microorganisms mostly due to the lack of highly enriched cultures. For a summary on the laboratory enrichments and environmental detection of N-dAOM microorganisms since 2006, culture conditions, inoculum sources, and environmental detection see Ding and Zeng (2021). In the following sections, an overview on the recent discoveries relevant to application is given highlighting the research gaps that need further investigation to point N-dAOM research in the right direction towards becoming a full-scale application.

#### Nitrite and Nitrate as a Central Link for Anaerobic Ammonia- and Methane-Oxidizers

Nitrite is the common terminal acceptor for Anammox and *Methylomirabilis* bacteria to oxidize ammonia and methane respectively (Figure 7; Strous et al., 1999a; Ettwig et al., 2010), and is the intermediate byproduct during nitrate reduction coupled to methane oxidation by *M. nitroreducens* (Figures 3, 7; Gambelli et al., 2018). This has been corroborated in several laboratory enrichments where nitrite was the defining factor between the co-existence of both N-dAOM microorganisms with anammox bacteria (Ding et al., 2014; Stultiens et al., 2019). The competition for nitrite is dependent on the affinity, whereas inhibition is subject to the formation of nitrite from ammonia oxidation (Figure 7) and is the main engineering parameter that can influence the out-competition of *Methylomirabilis* bacteria as anammox bacteria have a higher affinity for nitrite (Table 1; Winkler et al., 2015). Inhibition would originate from a surplus of nitrite formation under an excess in oxygen supply. *Methylomirabilis* bacteria have a tolerance of approximately 500 μM of nitrite (Guerrero-Cruz, 2018) whereas anammox bacteria have a tolerance ranging in the mM range (>10-fold higher) (Lotti et al., 2012; Table 1).

**TABLE 1:** Summary of relevant physiological parameters of both anaerobic ammonia and methane-oxidizing microorganisms.

	**Anammox bacteria**	***Methylomirabilis* bacteria**	***Methanoperedens* archaea**
Doubling time (days)	10 – 12^b^ 11^a^ 3^f^	25^e^ 14^c^ 5^i^	14–21 d at SRT of 10 d^–1–h^ (40% archaea enrichment)
μMax(d^–1^)	0.33^f^	0.0495^g^ 0.14^i^	ND
Yield (C-mol/energy source)	0.066 ± 0.01^a^	0.077 ± 0.027^i^	ND
Methane affinity	NA	92 ± 5 μM^e^	>1000 μM^d^ (S-dAOM)
Methane V_*max*_	NA	97 μM^e^ 2.6 ± 0.7 ± μM^i^	ND
Ammonia affinity	<5 μM^a^	NA	NA
Nitrite affinity	0.2–5 μM^b^	910 ± 90 μM^e^ 7 μM^i^	ND
Nitrate affinity	NA	NA	150 ± 29 μM^j^
Nitrite inhibition	400 gNO_2_-N m^3b^	40 g NO_2_-N m^3e^ Estimated at 500 μM^h^	Estimated at 500 μM^h^

*Values from both real enrichments and mathematical models (underlined) are shown, NA = not applicable, ND = not described. All data has been collected from ^*a*^Strous et al. (1998); ^*b*^van der Star et al. (2007); ^*c*^Ettwig et al. (2009); ^*d*^Meulepas et al. (2009); ^*e*^He et al. (2013); ^*f*^Lotti et al. (2015); ^*g*^Winkler et al. (2015); ^*h*^Guerrero-Cruz (2018); ^*i*^Guerrero-Cruz et al. (2019); ^*j*^Lu et al. (2019).*

#### Growth of N-dAOM Microorganisms

Growth rates, doubling times and yield (the amount of biomass obtained from the energy source) of N-dAOM microorganisms are lower compared to canonical aerobic heterotrophic microorganisms. N-dAOM microorganisms have doubling times estimated from weeks to months based on the quantification of 16S *rRNA* gene over time from environmental studies and long-term incubations (Ettwig et al., 2009; Vaksmaa et al., 2017). Recently, from a limited number of highly enriched cultures of N-dAOM microorganisms, data on growth and substrate kinetics has become available. *Methylomirabilis* bacteria can double every 5 days under maximum SRT conditions (10 days) and exhibit an estimated yield at steady state of 0.077 ± 0.027 C_mole_ CH_4 mole_^–1^, as determined by indirectly comparing the expected biomass decay versus the total biomass content while monitoring side populations through fluorescent microscopy (Guerrero-Cruz et al., 2019). For *“Ca. M. nitroreducens”* indications of a doubling time between 14–21 days have been obtained under continuous bioreactor incubation under an SRT of 20 days (Guerrero-Cruz, 2018). For comparison, aerobic methanotrophs generally exhibit a doubling time of ∼30 min to hours (Ogiso et al., 2012). Anammox bacteria have reported doubling times as low as 3 days and a biomass yield of 0.066 ± 0.01 C-mole/ammonium mole (Strous et al., 1998; van der Star et al., 2007). Table 1 gives a summary of relevant parameters in literature for anammox bacteria and N-dAOM microorganisms.

Another feature crucial to microbial growth is the capability to form granular biomass structures. Granular growth is essential to ensure the decoupling of SRT from HRT, preventing biomass washout. In addition, granular growth enables a multi-layer growth in oxygen limited systems resulting in the compartmentalization where, anaerobic microorganisms are protected at the core of the granular unit and aerobic bacteria secure the consumption of oxygen in outer layers (Winkler et al., 2015; Speth et al., 2016). Granular growth held a game changing role in the implementation of novel technologies relying on multiple processes occurring simultaneously such as in AD, NEREDA^®^, and PN/anammox (Lettinga et al., 1980; Lackner et al., 2014; Pronk et al., 2015; Chen et al., 2020; Trego et al., 2020).

*Methanoperedens* archaea and *Methylomirabilis* bacteria have the natural capacity to develop granular growth under dynamic conditions in SBR reactors (Luesken et al., 2011a; Guerrero-Cruz et al., 2019). Under continuous stirring in a membrane bioreactor, *Methanoperedens* archaea achieved higher enrichment levels in a granular ultrastructure from a sediment inoculum (Gambelli et al., 2018; Guerrero-Cruz, 2018). With the relevance of granular growth and the potential to form multi-layered microbial processes under oxic conditions, the next section describes the current knowledge of the oxygen effect on these microorganisms.

#### Oxygen and N-dAOM Microorganisms

*Methanoperedens* archaea perform the reverse action of methanogenesis and the McrA complex is highly sensitive to oxygen (Thauer, 1998). As such, *Methanoperedens* has been enriched under strict anoxic conditions. Recently, it was demonstrated that an enriched culture of *Methanoperedens* archaea (83% of the total community) expressed the genetic capacity to counteract the adverse effects of oxygen-stress upon 5% v/v oxygen exposure (Guerrero-Cruz et al., 2018). Although further research is needed to characterize how the recovery after oxygen exposure would occur, especially in natural environments. The resent findings corroborate that under oxygen-limited conditions *Methanoperedens* archaea could adapt to partial aeration most specifically in multi-layered granular growth.

*Methylomirabilis* bacteria are inhibited by oxygen exposure as low as 2% (Luesken et al., 2012) and have demonstrated the capacity to recover methanotrophic and nitrite reduction capacity after exposure to <1.1% oxygen (Kampman et al., 2018). The successful implementation of N-dAOM is subject to oxygen exposure in engineered ecosystems under high hydraulic dynamics conditions, however, the combination with oxygen-limited systems where aerobic ammonia oxidizers consume oxygen for the production of nitrite could be a suitable coupling under granular microbial growth.

#### Metals and N-dAOM

Metals are essential for microbial activity and growth, aerobic methanotrophs and anaerobic *Methylomirabilis* bacteria are not an exception, requiring copper for the catalytic activity of the pMMO enzyme (Bollinger, 2010; Ho et al., 2013b; Semrau et al., 2018). Recent studies provide direct evidence of the effect of copper on methane oxidation by *Methylomirabilis* bacteria, in laboratory enrichments a concentration of 5–6 micromolar of copper salt in the trace elements, triggered an increase in methane oxidation rates (Hatamoto et al., 2018). Furthermore, higher concentrations of copper and other metals seem to enable higher enrichment ratio of these difficult to cultivate bacteria (Guerrero-Cruz et al., 2019). Besides copper, lanthanide metals are also relevant for the intra-aerobic *Methylomirabilis* methanotrophs such as the novel enriched species *“Ca. Methylomirabilis lanthanidiphila”* and its uncultured environmental analog “*Ca. Methylomirabilis limnetica”* encoding for only a lanthanide-dependent MDH in a way that is not redundant as the first species reported in 2010 (Graf et al., 2018; Versantvoort et al., 2018). This change in MDH enzymes was discussed previously in this review (Section “Anaerobic Methanotrophs, Physiology Dictates Prevalence”), but the concentration of cerium in the trace elements during cultivation is a noteworthy factor. Moreover, iron is perhaps one of the most essential metals in life enabling formation of complexes and a wide spectrum of redox reactions that are crucial to enzymatic processes (Liu J. et al., 2014). Iron is present in cytochrome proteins, essential and highly abundant in different *Methanoperedens* archaea (Arshad et al., 2015; Leu et al., 2020b). Furthermore, increasing iron concentrations has resulted in higher laboratory scale enrichments. Initial attempts to enrich *Methanoperedens* archaea included iron concentrations below 5 micromolar (Vaksmaa et al., 2017), and concentrations of 80 micromolar have resulted in a two-fold increase in enrichment under laboratory scale conditions (Guerrero-Cruz et al., 2018; Lu et al., 2018). Evidently, metals (e.g., copper, cerium, and iron) are crucial for the activity and enrichment of anaerobic N-dAOM microorganisms. To date limited studies have directly compared the effect of trace metal concentrations in enriching these microbes, potentially due to low concentrations in the nature and lack of bioavailable forms. The future applications of these microorganisms must thus account for the role of metals in mix cultures and under varying conditions anticipated in wastewater treatment.

#### Methane Supply and Reactor Configuration

Methane has a relatively low water solubility (0.03464 Bunsen coefficient at 20°C or 1.237 mM at standard conditions, Wiesenburg and Guinasso, 1979; Haynes, 2011). The poor solubility of methane in effluents from wastewater treatment is a major contributor to methane emissions (Section “Wastewater Treatment as a Source of Methane Emissions”), and this translates to laboratory conditions where the supply of methane is the main limitation for the development of enrichment cultures. Diverse forms of bioreactor design have been implemented to ensure sufficient supply of methane, such as high methane flow, pressurized reactors, membrane-fed reactors or batch incubations. Table 2 provides a summary of relevant configurations in literature. It is important to note that due to the slow growth of N-dAOM microorganisms, most enrichments are achieved after months or even several years. Furthermore, these enrichments typically are achieved in bioreactors, with volumes not exceeding 10 L.

**TABLE 2:** Summary of diverse laboratory-scale bioreactor studies combining N-dAOM microorganisms under different bioreactor conditions.

**Reactor type**	**Microbial community**	**Performance**	**Limitation**	**Reference**
Membrane biofilm reactor (MBfR). Methane supply at 2 atm Oxygen 100 to 700 mL d^–1^ (50 d period).	Derived from Haroon et al. (2013).	1.5 Kg N m^3^d^–1^ (98% removal efficiency) Methane removal was not the focus.	External methane supply (95%).	Liu et al., 2019b
Membrane Granular Sludge Reactor Methane supply at 0.1 atm.	N-dAOM archaea (32%) and bacteria (9%), with anammox (27%) approximately.	16.53 kg N m^–3^ d^–1^ Methane removal was not the focus.	External methane supply (95%).	Fan et al., 2019
Membrane biofilm reactor (MBfR), increase methane pressure 1.44 atm.	N-dAOM archaea and bacteria, with anammox, proportions similar to Haroon et al., 2013.	≈ 2-fold N-dAOM archaea activity increase (26.1 mM N d^–1^). ≈ 5-fold N-dAOM bacteria activity increase (41.4 mM N d^–1^). Methane removal was not the focus.	External methane supply (90%).	Cai et al., 2018a
Membrane bioreactor (MBR) 19–25 mg methane L^–1^ 35.7 mg NH_4_^+^ L^–1^.	Not accurately characterized.	60% nitrogen removal in anaerobic conditions 95% methane removal.	Methane from the effluent of an UASB reactor.	Sánchez et al., 2016
Membrane biofilm reactor (MBfR). Methane at a flow from 0.1 to 0.5 atm.	N-dAOM archaea (74.3%) and bacteria (11.8%), with anammox (5.6%).	1.3 mM nitrate d^–1^ and 2.1 mM d^–1^ ammonia. Methane removal was not the focus.	External methane supply (95%).	Fu et al., 2017
Membrane biofilm reactor (MBfR). Methane supply at 2.46 atm.	N-dAOM archaea (50%) and bacteria (20%), with anammox (20%).	48.8 mM N d^–1^ overall. (1.2 Kg N m^3^d^–1^) Methane removal was not the focus.	External methane supply (95%).	Xie et al., 2017
Membrane biofilm reactor (MBfR).	N-dAOM archaea (50%) and bacteria (20%), with anammox (20%).	48.8 mM N d^–1^ overall. Methane removal was not the focus.	External methane supply (90%).	Cai et al., 2015, derived from Shi et al. 2013
Sequencing batch reactor, batch methane pressure 0.4 Mpa.	N-dAOM archaea (29%) and bacteria (12%), with anammox (21%).	4.84 mM d^–1^ Nitrate Methane removal was not the focus.	External methane supply (95%).	Ding et al., 2014
Membrane biofilm reactor (MBfR).	N-dAOM archaea (20–30%) and bacteria (20–30%), with anammox (20–30%)	13.6 mM N d^–1^ Methane removal was not the focus.	External methane supply (90%).	Shi et al., 2013

Laboratory scale membrane bioreactors (MBR) have been proposed as an ideal setup for the enrichment of N-dAOM microorganisms, usually accompanied by anammox bacteria (Table 2). MBRs ensure methane supply and biomass retention, however, relying on the external supply of artificial high-purity methane mixtures (>95%), which are costly. Pressurized systems have been used to enrich S-dAOM methanotrophic archaea and have demonstrated to enable higher growth and activity rates by ensuring the availability and saturation of methane above normal conditions of methane (Nauhaus et al., 2002; Timmers et al., 2015). The combination of pressurized reactors and membrane configurations has indeed shown an increase in the activity of N-dAOM microorganisms (Cai et al., 2018a), and these studies highlight the application potential, however the external supply of high-purity methane remains the main obstacle for full-scale application in WWT. When high-quality external supply of methane is required, the potential use of intrinsically-produced methane from AD is overlooked and deviates from the prospective application of N-dAOM microorganisms to mitigate GHG emissions from effluents of AD in WWT (Wang et al., 2017), where commonly a dual treatment configuration is proposed with partial PN/A and N-dAOM as a second step or in combination with anammox (Stultiens et al., 2019). Moreover, membrane-based reactors provide a suitable surface for biofilm growth (Cai et al., 2018a) as opposed to granular-based growth (Guerrero-Cruz et al., 2018). Thus, the stratification proposed as crucial factor for the implementation of anaerobic processes under micro-aerophilic conditions is likely to not occur (Winkler et al., 2015). Furthermore, studies where oxygen exposure at a constant level of 0.01 mg L^–1^ have shown no negative effects on N-dAOM/partial nitritation/anammox systems (Liu et al., 2019b), but the small scale does not anticipate the effects under high volume of effluent input in real applications.

In this section, relevant aspects affecting the performance and growth of N-dAOM microorganisms were described. Currently the advancement of N-dAOM-based technology is faced with bottlenecks in the following aspects:

(i) Most work relies on methane-rich artificial gas mixtures (>90 percent). There are no studies with real wastewater, except for one study where in a 50 L pilot plant, dissolved methane was removed (95% removal, 78% methane-containing biogas from a UASB reactor at a rate of 31 L per day) coupled to denitrification (1.43 mM total nitrogen removal). However the role of N-dAOM microorganisms in a dual post-treatment containing oxic and anoxic compartments was not fully characterized next to the use of synthetic media (Silva-Teira et al., 2017). The bioaugmentation of biogas, and removal of toxic sulfur compounds could improve the use of waste biogas to enable the application of N-dAOM microorganisms when not suitable for electricity production.
(ii) Studies to optimize fundamental aspects of growth and enrichment conditions are lacking. Currently, evidence on the role of iron, copper, and lanthanide is available (Hatamoto et al., 2018; Lu et al., 2018; Guerrero-Cruz et al., 2019); however, further studies on the requirements of these micronutrients, including up-scaling and modeling aspects in large-scale applications, are still lacking. The availability of these metals could improve the activity, growth, competitiveness, and enable the application of N-dAOM microorganisms in oxygen-limited WWT systems.
(iii) Methane supply and the gas-liquid transfer are crucial for the successful implementation of N-dAOM microorganisms where *Methylomirabilis* bacteria compete with aerobic methane oxidizers in regard to their methane affinities (Guerrero-Cruz et al., 2019), *Methanoperedens* archaea would need the assistance of pressurized systems to increase methane saturation above 1.3 mM and reach an equilibrium for the McrA complex to perform methane oxidation at sustainable rates (Cai et al., 2018a).
(iv) Bioreactor configuration is a limiting factor, where membrane-based bioreactors rely on biofilm formation rich in N-dAOM microorganisms which lacks spatial niche differentiation (Cai et al., 2018a; Liu et al., 2019b). This prevents the formation of compartments that allow for both aerobic and anaerobic processes to co-exists as in the case for granular growth (Winkler et al., 2015; Gambelli et al., 2018; Guerrero-Cruz et al., 2018). Furthermore, biofilm formation is a limiting phenomenon in large scale-membrane systems whereas granular-based bioreactors have successfully been implemented in large-scale applications worldwide (Lettinga et al., 1980; Lackner et al., 2014). A combination of granular-based N-dAOM microorganisms with adequate methane-transfer adaptation through membrane systems or pressurized reactors, could offer significant advantages to apply the N-dAOM process.

After highlighting the diverse factors affecting the performance of N-dAOM microorganisms and knowledge gaps in the research, it is evident that reactor configuration and more importantly the synergistic combination with other processes are crucial research questions. Exploring these aspects can reveal the true value whether N-dAOM microorganisms could be applicable and biotechnologically beneficial. Anaerobic microbes and processes in anoxic compartments are undoubtedly applicable under granular configurations where both aerobic and anaerobic processes occur simultaneously in WWT (Pronk et al., 2015; Winkler et al., 2015). Thus research on complex microbial ecology and interactions is needed for the realization of more integrated WWT technologies. Moreover, diverse operational challenges such as aeration resulting in gas stripping, play a crucial role in the release of methane into the atmosphere. To circumvent these aspects, membrane-based and/or pressurized systems have been discussed as a means of enabling methane-rich conditions for the implementation of N-dAOM microorganisms. Currently, there is no “one fits all” solution that fully integrates all the aspects that challenge the application of N-dAOM from both points of view: microbial ecology and the engineering limitations. Nevertheless, the collection of high quality biogas for membrane-based configurations or biofilm-based oxic/anoxic systems; remain as potential solutions (Liu et al., 2019a). Indisputably, biomass stratification and the controlled supply and retention of methane in the system, are crucial requirements for the coexistence of both aerobic and anaerobic processes (Liu et al., 2019b). Moreover, efficient biogas collection and bioaugmentation are important steps to secure high-quality biogas feed for integrated systems that aim to remove methane and nitrogen contaminants simultaneously where biogas is otherwise not fitted for heat and electricity production. The next section describes alternative uses for methane-rich mixtures.

## Applications of Aerobic Methanotrophs in a Circular Economy

Currently natural gas is the primary source of methane for industrial and chemical production of methanol and other industrial compounds. However natural gas reservoirs are finite and is thus a non-renewable fuel source. Furthermore, the industrial production of methanol and olefines is not environmentally friendly, requiring large amounts of energy, metal catalysts, and high temperature and pressure conditions (Da Silva, 2016). Methanotrophic microorganisms are natural catalyzers that can perform many biochemical conversions at ambient temperatures and without the need of high energy input compared to industrial processes, making their potential applications relevant sustainable alternatives (Wang et al., 2020). Biogas from AD, is a low-value feedstock for new technologies that harness the potential of methanotrophic bacteria to produce valuable compounds such as methanol, formaldehyde, or short chain fatty acids, and biopolymers (Comer et al., 2017).

**FIGURE 8:**
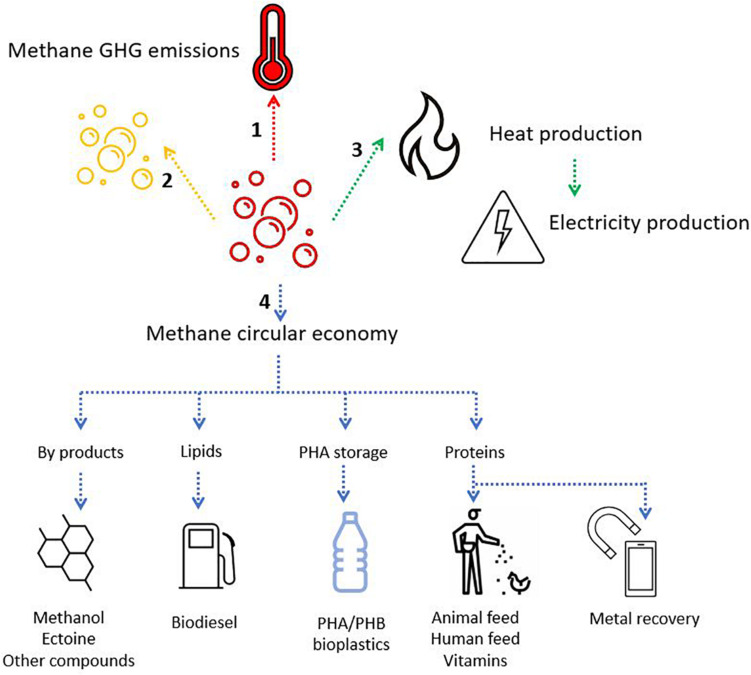
Schematic representation of the fate of methane. (1) No action leads to methane-GHG release to the atmosphere (red arrow). (2) Methane can be neutralized into CO_2_, a less harmful GHG (in a short timescale) (yellow arrow). Methane oxidation can occur aerobically or anaerobically depending on the availability of electron acceptors and the microorganisms present. (3) Methane can be collected and used as a biogas for heat and electricity production (green arrow). (4) Methane can enter bio-based and circular economy models through the application of aerobic methanotrophs for the production of various chemicals, biodiesel, bioplastics, protein, and even the potential recovery of precious metals (blue arrows).

The fate and use of methane can follow several paths, and depends on infrastructure, policy, and the adoption of a waste-to-value culture (Figure 8). The production of valuable compounds from methane by methanotrophs has three differentiable targets: (i) Methanotrophs produce methanol as a valuable intermediate compound; (ii) Methanotrophs produce biopolymers as a carbon storage molecule; and (iii) Methanotrophs utilize methane as the building block for cell constituents (lipids or proteins). The application of aerobic methanotrophs for bioremediation and as bio-catalysis for a range of compounds can be found in Strong et al., 2015. Moreover, capitalizing on novel mechanistic insights of the aerobic methanotrophs under oxygen-limiting conditions, the methanotrophs can potentially be applied to produce volatile organic compounds (e.g., acetate, lactate; Kalyuzhnaya et al., 2013), and in methane-driven bioelectrochemical systems (Zheng et al., 2020). Accordingly, aerobic methanotrophs may also drive denitrification in WWTP, either in synergistic interaction with other microorganisms being a source of electron donor (e.g., by excreting acetate under oxygen limitation; Costa et al., 2000) or as a coping strategy under hypoxia (Kits et al., 2015; Zhu et al., 2016). Hence, emerging applications of aerobic methanotrophs may rely on their metabolic capability under oxygen limitation, but this aspect of methanotroph biotechnology needs further exploration. Here, we focus on the role of aerobic methanotrophs under predominantly oxic conditions in the production of value in the form of biopolymers and two examples of valuable chemical intermediates with current proof of principle and with proven potential at industrial scale.

### Methane as Energy Source and Chemical Building Block

#### Methanol

Methanol for industrial use is mainly produced from natural gas and to a lesser extent from CO_2_ exhaust waste streams through unsustainable thermochemical processes, at a rate of 110 million metric tons per year. Methanol is one of the most important building blocks for nearly 50000 different chemicals. Approximately one third of it is used for formaldehyde production, followed by synthesis of olefins for the plastic industry (Da Silva, 2016). Biogenic methane from anaerobic digestion could be a source for methanol production through the application of methanotrophs when methane-rich biogas for electricity production is rendered non cost-effective in the near future.

Aerobic methanotrophs oxidize methane to methanol, and its recovery requires the inhibition of the further methanol oxidation step. Ammonium chloride, phosphate, cyclopropanol, CO_2_, NaCl, and EDTA are known MMO inhibitors that could potentially aid in the recovery of methanol from methanotrophic cultures (Wang et al., 2020). For a summary of culturing and recovery strategies see Bjorck et al. (2018). *Methylosinus trichosporium* OB3b, is the most extensively studied methanotroph for methanol production using Na-formate and NaCl to inhibit MDH activity reaching 7.7 mM of methanol in 36 h (Sang et al., 2004) or cyclopropanol with methanol levels of 152 mmol g^–1^ DW cells with a methanol conversion efficiency of 61% (Takeguchi et al., 1997). Regarding methane conversion efficiency in *M. trichosporium* OB3b, studies have reported 64% (Duan et al., 2011) and 80% yields but the production of methanol at higher efficiency remains a challenge (Han et al., 2013). A species from the genus *Methylocaldum*, was reported to produce methanol from methane-biogas upon 500 ppm of hydrogen sulfide exposure in batch cell suspensions, with a methane conversion efficiency of up to 34% and methanol yields of up to 343 mg L^–1^ over 105 h incubations (Zhang et al., 2016). Although the application of methanotrophs for the production of methanol seems promising based on laboratory scale evidence, industrial applications are unlikely at this stage due to the need of external electron donors during MDH inhibition, e.g., formate (Sheets et al., 2016), negatively affecting the feasibility of large-scale applications and the continuous performance of methanotrophs.

Proteobacterial methanotrophs have been widely studied, however their mesophilic nature and implementation in non-sterile conditions are still a bottleneck. Verrucomicrobial methanotrophs, are known to thrive at low pH and extremely high temperatures, making them a promising alternative; however, the feasibility to produce methanol from Verrucomicrobia has not been investigated in depth. Attempts to produce methanol through the inhibition of MDH’s requires external supply of electron donors and growth conditions under multiple inhibition strategies, rendering the application of these extremophiles unclear at the low pH these bacteria are known to inhabit (Hogendoorn et al., 2020). Nevertheless, the exploration of Verrucomicrobial strains for methanol production from biogas, could be advantageous when using biogas mixtures that contain hydrogen sulfide as canonical proteobacterial methanotrophs are known to be intolerant to this toxic byproduct of AD biogas (Börjesson, 2001). Another feature of interest in using Verrucomicrobial methanotrophs is the possibility to reduce culture contamination by having operating temperatures as high as 55 degrees and/or lower pH values (Chen and Jiang, 2018). Research in efficient methanol recovery without a negative impact on microbial growth that could compromise culture yields, is crucial to explore biological methanol production, however, remains to be the bottleneck due to metabolic constraints for cellular growth and energy conservation.

#### Ectoine

Ectoine and hydroxyectoine are molecules produced in response to osmotic stress in numerous bacteria, their chemical properties protect cells during osmotic stress and assist stabilization and preservation of cell integrity without altering cell function. These properties result in a wide spectrum of applications in the cosmetic industry, medicine, dermatology, nutrition, and pharmaceuticals as well as in preservation products for a broad range of cell biology applications (Pastor et al., 2010; Wang et al., 2020). Ectoines are valued at one thousand United States dollar per kilogram, and the current global need is 15 million Kg a year, representing the most profitable market at 15 billion United States dollars a year (Strong et al., 2016). Among the methanotrophs, *Methylomicrobium alcaliphilum* shows the highest reported accumulation of ectoine, at 8.3% of ectoine per gram of biomass (Cantera et al., 2017). Heterotrophic bacteria, however, showed 3 orders of magnitude higher ectoine production rates than methanotrophs (Salar-García et al., 2017). Some of the factors that affect the efficiency of methanotrophs to accumulate ectoine is the susceptibility to mechanical stress in culture enrichments (Cantera et al., 2016). Also, the presence of specific metals is crucial for enzymatic performance. To this end, *Methylomonas* has shown increased activity in the presence of copper, enhancing ectoine production (Cantera et al., 2016). With the significant financial market, the use of methanotrophs seems like a suitable application using methane waste streams while enabling the creation of value from waste.

### Methane as Source for Microbial Biopolymers

#### Biodiesel From Methane-Derived Lipids

Biodiesel production has gained attention as a more environmentally friendly alternative to diesel, currently produced from diverse crops in marginal lands, with particular environmental debate over the use of land in competition with food agriculture [Intergovernmental Panel on Climate Change (IPCC), 2012]. Conversely, biodiesel can be produced from membrane lipids of methanotrophic bacteria. Methanotrophs, in comparison to other heterotrophs, have an increased lipid content due to the stacked internal membranes, rendering them attractive for biotechnological applications after a culture fed with methane has reached optimal growth yields to secure the maximum ratio of biomass from mol of methane.

*Methylomicrobium buryatense* 5GB1, a Type I methanotroph, is used as the model microorganism for the production of membrane lipids. Typical to Type I methanotrophs, this microorganism assimilates carbon through the RUMP pathway and fatty acids are produced from Acetyl-CoA (Wang et al., 2020). Up to 95% of the total fatty acids from membrane-bound phospholipids were recovered in *Methylomicrobium buryatense* corresponding to a total fatty acid content of 10% of dry cell weight achieved in batch reactors (Dong et al., 2017). Furthermore, a glycogen knockout strain AP18, has shown to have a higher lipid yield due to the suppression of glycogen production leading to a better feedstock for lipid conversion to biodiesel (Fei et al., 2018). Fatty acids are then extracted using an alkaline hydrolysis at 150°C followed by a hexane extraction where recoveries can reach up to 95% of the methanotrophic culture’s total lipids. Lastly, a chemical upgrading of the fatty acids takes place using palladium catalysts for the hydrodeoxygenation process that transforms the lipids into long chain hydrocarbons rich in pentadecane (up to 88% efficiency) (Furimsky, 2000). Chemical upgrading of methanotrophic lipids is essential to remove reactive oxygen (carbon saturation) from the lipid molecule, conveying less reactivity and viscosity, and increasing the calorific capacity of the final product.

#### Bioplastics

Polyhydroxy alkanoates (PHAs) have become an important biopolymer in the past years as a replacement to chemically produced plastics (Amaro et al., 2019). PHA is a collective term encompassing approximately 100 types of monomeric compounds, all of them being linear polyesters with different degrees of polymerization reaching up to 30 thousand units. Under the right conditions, several types of bacteria can accumulate up to 90% of PHA making bacteria-derived PHA a cost-effective strategy to produce bioplastics (Madison and Huisman, 1999).

Several microbes such as *Haloferax mediterranei* and *Alcaligenes latus* produce high PHA levels, reaching more than 50% of the cells’ dry weight. Genetically modified *Escherichia coli* expressing PHA biosynthesis genes (*phbA*, *phbB*, and *phbC*), accumulate PHA from lactose (Park et al., 2001). However, the feedstocks used in the production of PHAs from engineered microbes are expensive and rely on glucose and other carbohydrates, posing additional concerns regarding the sustainable supply chain, and land use for crop production which competes with crops destined for human consumption. Methane is a naturally occurring carbon source for microbial production of PHAs, and some methanotrophic bacteria are efficient at accumulating PHA without the need for genetically engineered metabolism. The possibility to use a cheap carbon source through the application of bacteria to produce bioplastics while mitigating methane emission, broadens the use of methanotrophs in a circular and bio-based economy. Bioplastics produced from poly-3-hydroxy butyrate-co-3-hydroxyvalerate [P(3HB-3HV)] can be degraded within weeks to a few years depending on the environmental conditions. These timescales are significantly lower than for non-biodegradable plastics such as polypropylene (Mergaert et al., 1994). Diverse products have been manufactured using PHA, for a summary with numerous patents with inventions from P(3HB-3HV) see Madison and Huisman (1999).

The accumulation of PHAs in methanotrophs is a survival response under nutrient starvation which re-routes methane into PHA production instead of converting it to carbon dioxide under normal conditions (Karthikeyan et al., 2015). In nature, the accumulation of PHA occurs under fluctuating nutrient (nitrogen, carbon) availability. In proteobacterial methanotrophs, only Type II are known to possess the genetic capability to express enzymes that produce PHA and contain pathways to direct the TCA cycle into PHA production (Pieja et al., 2011a). The genes *phaA*, *phaB*, and *phaC* encode for the enzymes that perform the condensation of two molecules of Acetyl-CoA, their reduction into (R)-3-hydroxybutyl-CoA, and finally the polymerization into the final PHA molecule, respectively (Peoples and Sinskey, 1989).

Overall, the growth conditions that lead to the accumulation of PHAs are: (i) absence of ammonia or nitrate as nitrogen source, (ii) copper deprivation and (iii) a regime of fed-batch to induce storage of carbon in the cell. *Methylocystis* sp. Rockwell, *Methylocysti*s sp. WRRC1, *Methylosinus trichosporium* OB3b, *Methylomicrobium album* BG8, and *Methylomonas denitrificans* FJG1 have been subjected to studies aiming to determine the preferred nitrogen (ammonia or nitrate) and carbon (methane or methanol) source (Tays et al., 2018). In general ammonia and methane are preferred as nitrogen and carbon source, respectively; however, the in-depth characterization of each strain is crucial before the application for industrial use where maximum yields, minimum inhibition and maximum efficiency of product are needed. Using a combination of continuous culture, followed by nitrogen limitation under fed-batch has proven to be successful in the enhancement of PHB accumulation in *Methylocystis* sp. GB25 DSM 7674, reaching 51% of cell mass as PHB (Wendlandt et al., 2005). Another key regulator are metals, in particular lanthanides which modulate the activity of the MDH, and play a crucial role in the carbon flow within the cell which have implications on the routes to carbon storage in the form of PHA (Akberdin et al., 2018). Inherent to the vast diversity of methanotrophs, nutrient conditions and in particular micronutrients, require finetuning according to the metabolic needs of particular strains. Table 3 provides a summary of those requirements in individual culture cases.

**TABLE 3:** Summary of selected laboratory enrichments and the conditions involved for the production of PHA in methanotrophic bacteria.

**Organism**	**Cultivation condition**	**Effect**	**Reference**
*Methylocystis* sp. GB25 DSM 7674	Enriched culture (86%) Potassium deficiency.	45% PHB yield per g of methane.	Helm et al., 2008
*Methylocystis* sp. GB25 DSM 7674	Continuous and fed-batch alternation, nitrogen limitation.	55% PHB yield per g of methane.	Wendlandt et al., 2005
*Methylocystis parvus OBBP*	Reducing substrates methane and formate	Enable PHB utilization as carbon source.	Pieja et al., 2011b
*Methylocystis* sp. WRRC1	Flasks cultures with Valerate or n-propanol additions. Copper deprivation conditions.	Decrease in melting temperature and crystallinity of PHB-*co-*HV 67% PHB per g of methane.	Cal et al., 2016

*For more reports on PHB efficiencies, see Pieja et al. (2017).*

The use of augmented biogas from wastewater is already applied at full-scale (Mango Materials^®^). However, more research on lower quality biogas use is needed to exploit emerging opportunities with biogas as a substrate while mitigating GHG emissions from waste management.

#### Single Cell Protein (SCP) From Methanotrophs

Another methanotroph-derived biopolymer includes proteins from single cells (SCP). SCP can be purified from all methanotrophs as all carbon fixation pathways yield structural proteins that form new cells during microbial growth, and its use dates back to the early 1960s as the first bio-based product from methane (Bewersdorff and Dostálek, 1971).

All proteins are formed by polymers of amino acids and depending on their quaternary structure and function, harbor cofactors such as vitamins and metals, making them a suitable feed source for diverse animals regardless of their origin (Skrede et al., 1998, 2003; Øverland et al., 2010). Proteins of microbial origin have better yields compared to other protein sources and offers comparable nutritional quality when used as feed according to FAO recommendations (Anupama and Ravindra, 2000; Ritala et al., 2017). The expanding population growth and the need to feed nearly 10 billion people by 2050 (FAO, 2009), demand for sustainable protein sources. These are crucial in replacing the massive exploitation of marine and freshwater resources where the increase in aquaculture is a recent example of a sustainable practice that still requires significant amounts of protein as feed for produce (Ritala et al., 2017).

Ideal SCP methanotrophic strains should have a fast growth rate with high protein production capacity and easy to cultivate. Furthermore, the methanotrophs should be able to tolerate a wide range of pH and temperatures. The use of methane from natural gas for the production of SCP, is already approved for animal feed in agriculture (Wang et al., 2020). Currently SCP is produced from methane-rich natural gas and commercially applied at an industrial scale (Strong et al., 2015). Other sources deemed ideal for the production of SCP include a variety of solid and liquid waste streams (Wang et al., 2020), whereas biogas from AD remains overlooked.

A challenge for the successful application of methanotrophs is the poor solubility of methane in the aqueous phase (Bennett et al., 2018). Different reactor configurations that enhance the mass transfer have been tried successfully at a laboratory scale. Petersen et al. (2017) used a forced flow U-loop reactor to study the effects of gas transfer and reported higher efficiencies when compared to canonical stirred tank reactors (STR) or even tubular reactors (Petersen et al., 2017). This application is available at a commercial scale using natural gas as a carbon source for the methanotrophs by UniBio A/S and Calysta Inc., for the production of UniProtein^®^ and FeedKind^®^ respectively. The methanotrophs used are commonly composed of a mix culture with unknown ratios. *Methylococcus capsulatus* or *Methylomonas* sp. as the most common strains described in the literature. For more examples of SCP from bacteria at an industrial scale, see Jones et al. (2020). Current trends are moving from methane-rich sources such as natural gas, to more circular economy approaches using waste streams such as wastewater. The concept of using co-cultures i.e., *Methylococcus capsulatus* with algae *C. sorokiniana*, has been proposed and research under conditions with supplementation of artificial waste biogas (60% methane) for SCP production showed promising results (Rasouli et al., 2018). Implementation of SCP production at a pilot-scale under real wastewater conditions has not been proven successful to date.

After the description of the relevance of anaerobic methanotrophs to counteract GHG emissions, and the role of aerobic methanotrophs in a bio-based and circular economy for a sustainable future, the value offered by methanotrophs seems immeasurable.

## Concluding Remarks

After nearly 120 years of discoveries, diverse (an)aerobic methanotrophs have been identified in widespread environments as key drivers of Earth’s carbon fluxes, playing a crucial role as the sole biological methane sink. Methanotrophs determine the fate of methane in both natural and human-impacted ecosystems, where the mitigation of greenhouse gas emissions is recognized as one of the most important environmental development goals. Importantly, methanotrophs have the potential to transform methane into valuable products.

Application of methanotrophs for both climate change mitigation and resource recovery in a circular economy face parallel challenges, namely limitations inherent to using methane as a substrate, and the need to revolutionize current chemical-based industrial practices to bio-based solutions. Past research on methanotroph ecology, physiology, and genomics yielded exciting discoveries. Future research direction on novel engineering solutions incorporating methane waste streams from human-influenced ecosystems (WWT), could lead to a new era of methanotrophic biorefineries, move waste management and production systems close to zero net emissions, and reconcile human intervention in the carbon cycle.

## Author Contributions

SGC, AV, and AH wrote the manuscript. MP, MAH, and HN contributed to the critical review and streamlining of the manuscript, SGC and AH coordinated the manuscript conceptualization. All authors contributed to the final version of the manuscript.

## Conflict of Interest

The authors declare that the research was conducted in the absence of any commercial or financial relationships that could be construed as a potential conflict of interest.
